# Why weight? Modelling sample and observational level variability improves power in RNA-seq analyses

**DOI:** 10.1093/nar/gkv412

**Published:** 2015-04-29

**Authors:** Ruijie Liu, Aliaksei Z. Holik, Shian Su, Natasha Jansz, Kelan Chen, Huei San Leong, Marnie E. Blewitt, Marie-Liesse Asselin-Labat, Gordon K. Smyth, Matthew E. Ritchie

**Affiliations:** 1 Molecular Medicine Division, The Walter and Eliza Hall Institute of Medical Research, 1G Royal Parade, Parkville, Victoria 3052, Australia; 2 Stem Cells and Cancer Division, The Walter and Eliza Hall Institute of Medical Research, 1G Royal Parade, Parkville, Victoria 3052, Australia; 3 Department of Medical Biology, The University of Melbourne, Parkville, Victoria 3010, Australia; 4 Bioinformatics Division, The Walter and Eliza Hall Institute of Medical Research, 1G Royal Parade, Parkville, Victoria 3052, Australia; 5 School of Mathematics and Statistics, The University of Melbourne, Parkville, Victoria 3010, Australia

## Abstract

Variations in sample quality are frequently encountered in small RNA-sequencing experiments, and pose a major challenge in a differential expression analysis. Removal of high variation samples reduces noise, but at a cost of reducing power, thus limiting our ability to detect biologically meaningful changes. Similarly, retaining these samples in the analysis may not reveal any statistically significant changes due to the higher noise level. A compromise is to use all available data, but to down-weight the observations from more variable samples. We describe a statistical approach that facilitates this by modelling heterogeneity at both the sample and observational levels as part of the differential expression analysis. At the sample level this is achieved by fitting a log-linear variance model that includes common sample-specific or group-specific parameters that are shared between genes. The estimated sample variance factors are then converted to weights and combined with observational level weights obtained from the mean–variance relationship of the log-counts-per-million using ‘voom’. A comprehensive analysis involving both simulations and experimental RNA-sequencing data demonstrates that this strategy leads to a universally more powerful analysis and fewer false discoveries when compared to conventional approaches. This methodology has wide application and is implemented in the open-source ‘limma’ package.

## INTRODUCTION

Second-generation sequencing technology provides researchers with a high resolution and cost-effective tool for surveying the complexity of the transcriptome in both health and disease. RNA-sequencing (RNA-seq) is now a routine tool for studying differential expression, alternative splicing, allele-specific expression and for discovering novel transcripts (1). In an RNA-seq ‘differential expression’ analysis, the goal is to determine which genes, transcripts or exons, show evidence for changes in expression between experimental groups relative to a realistic assessment of both technical and biological variation. In such analyses, the application of statistical modelling to remove systematic biases and reduce variability via normalization (2,3), batch correction (4,5) and subsequently prioritize changes in gene expression between treatment groups (6,7) have each been shown to play a key role in extracting meaningful insights from RNA-seq data.

**Figure 1.**
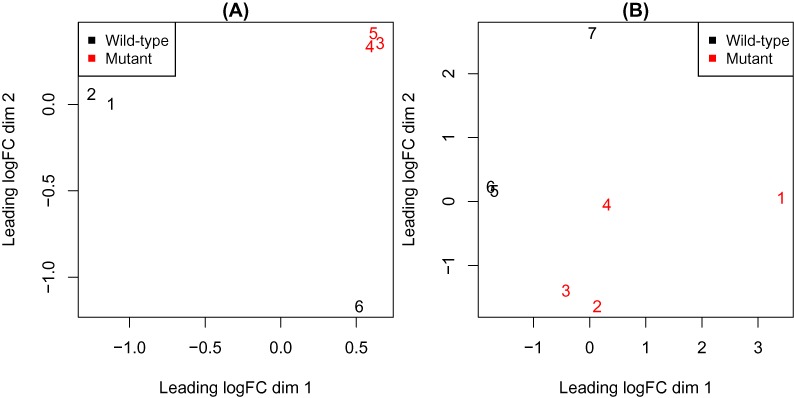
RNA-seq data sets where variations in sample quality are evident. Each panel shows a multi-dimensional scaling (MDS) plot, with samples colour-coded by experimental group. One or more samples that exhibit higher variability than average are present in each case ((**A**): sample 6; (**B**): samples 1 and 7). In these experiments, cells carrying a mutant allele of the gene *Smchd1* were compared against wild-type cells.

Variations in sample quality is another source of noise that makes the identification of differentially expressed (DE) genes more difficult. Sample-specific variation is often first observed by inspecting plots from a principal components analysis or multi-dimensional scaling (MDS). Figure 1 shows MDS plots from two RNA-seq experiments where a particular gene (*Smchd1*) has been mutated. In both experiments, one or more samples exhibit increased variation that is likely to be related to RNA quality (e.g. degraded RNA may be unavoidable in studies involving patient tissue) or sample purity. In our experience, sample quality variation is relatively common in RNA-seq data sets, and once identified, the researcher may choose to re-run the suspect samples or, in extreme cases, collect fresh samples. These options may not always be feasible due to resource constraints, therefore developing analytical methods that can address this variability and make the most of the data available would be advantageous.

There are currently no methods that can model sample-specific variation in small-scale RNA-seq experiments in a systematic way. One option is to remove samples with higher variability from the analysis. This has the benefit of reducing variation, but comes at the expense of reducing power, which can hamper our ability to detect DE genes. This approach assumes that samples with higher than average variation can be readily identified, which is not always the case. The decision of whether to keep or discard a particular sample is often based on *ad-hoc* cut-offs and a trial and error approach. At the other extreme, one could retain all samples in the analysis. In this situation, our ability to distinguish genuine differences between experimental conditions from the noise will be limited by the increased variation present. An intermediate approach would be to analyse the complete experiment but down-weight the observations from more variable samples, thus retaining the maximum degrees of freedom whilst discounting noisy observations.

Experience with microarray data has shown that this approach can be beneficial, with the use of quantitative quality weights at the sample-level offering improved results (8–10). Allowing for variations in quality between RNA samples is particularly important when samples are difficult to obtain such as when collected from human subjects or very rare cell types (11). RNA sample quality is typically related to the availability of tissue samples or cells and the ease of RNA isolation, a consideration that is relevant irrespective of whether microarray or RNA-seq is used to profile gene expression. The approach of Ritchie *et al*. (2006) (9) has allowed researchers to make discoveries that would otherwise have been missed, for example in phase I trials of cancer drugs (12).

We adapt this method to enable the use of quantitative quality weights in RNA-seq data sets to improve the results obtained in the presence of more variable samples. We model this variation using a combined approach that takes into account both global intensity-dependent trends in the variability using ‘voom’ (13) as well as sample-specific variability using a log-linear model that shares parameters between genes (9). Our new method is compared against a number of alternatives on simulated data, a control experiment where samples were mistreated to simulate increased variability and in a gene mutation study. Across a range of scenarios, we show that our combined observational and sample-specific weighting approach improves power for detecting known changes in gene expression and generates fewer false positives than other methods. This approach is widely applicable, suitable for use in most designed RNA-seq analyses and is available in the popular R-based ‘limma’ package (14).

## MATERIALS AND METHODS

### Linear models for RNA-seq differential expression

The entry point for our analysis is a matrix of counts that have been appropriately normalized and transformed into log_2_ counts per million (CPM) so that they are approximately normally distributed. The work of Law *et al*. (2014) (13) has shown that although the distributional assumption of normality is imperfect, the log_2_(CPM) transformation when combined with weights that take into account the unequal variabilities on this scale can lead to a more powerful analysis compared to methods that assume RNA-seq counts follow a negative-binomial distribution. Assuming normality allows us to model the variances, which we will exploit later. Variance modelling is not currently available for RNA-seq analysis using count distributions.

We begin by assuming a linear model where the systematic expression effects for each gene (or exon or other genomic feature of interest) can be described as

(1) \begin{equation*} E({\bf y}_g)=X {\boldsymbol \beta}_g, \end{equation*}

where **y**_*g*_ = (*y*_*g*1_, …, *y*_*gJ*_)^*T*^ is the vector of log_2_(CPM) values for gene *g*, *X* is a design matrix with full column rank and ***β****_g_* = (***β****_g_*_1_,…, ***β****_gK_*)*^T^* is a gene-specific vector of regression coefficients (15). The design matrix reflects the experimental design and choice of parameterization and the regression coefficients represent log-fold changes between RNA sources in the experiment. This model assumes

(2) \begin{equation*} {\rm var}(y_{gj})=\sigma ^2_{g}/w_{gj}, \end{equation*}

where $w_{gj}$is an observational level weight derived from the ‘voom’ model (13) for gene *g* in sample *j* and $\sigma ^2_{g}$ is an unknown factor.

If we assume that the *y*_*gj*_ are normally distributed and that expression values from different samples are independent, the weighted least squares estimator of ***β****_g_* is

(3) \begin{equation*} \hat{{\boldsymbol \beta }}_g=(X^T\Sigma _g^{-1}X)^{-1}X^T\Sigma ^{-1}_g{\bf y}_g, \end{equation*}

where $\Sigma_g=diag(w_{g1},...,w_{gJ})$ is the diagonal matrix of prior weights. The moderated *t*-statistic for testing any particular $\beta_{gk}$ equal to zero is

(4) \begin{equation*} t_{gk}=\frac{\hat{\beta }_{gk}}{s_g^*\sqrt{c_{gk}}}, \end{equation*}

where $s_g^{*2}$ is the shrunken residual mean square (15) from the weighted regression and $c_{gk}$ is the *k*th diagonal element of $(X^T\Sigma _g^{-1}X)^{-1}$. This moderated *t*-statistic has *J* − *K* degrees of freedom, and allows genes to be ranked for differential expression based on their *P*-values from this distribution (adjusted for multiple testing), or testing can be performed relative to a chosen fold-change (FC) (16).

### Heteroscedastic models for genes and samples

The approach of Ritchie *et al*. (2006) (9) allows unknown variance factors to depend on the sample as well as on the gene,

(5) \begin{equation*} {\rm var}(y_{gj})=\sigma ^2_{gj}/w_{gj}. \end{equation*}

The variance factors $\sigma ^2_{gj}$ reflect the fact that the genes differ in variability and also that samples in the experiment may differ in quality in a way that increases or decreases the variability of all or most of the genes in a particular sample. The additive log-linear model

(6) \begin{equation*} \log \sigma ^2_{gj}=\delta _g+\gamma _j \end{equation*}

is the simplest model that ensures variability depends multiplicatively on sample quality. The constraint $\sum _{j=1}^J\gamma _j=0$ gives us $\sigma ^2_g=\exp \delta _g$ for the gene-wise variance factors and $\gamma_{j}$ represents the relative variability of each sample. A particular sample *j* will have $\gamma_{j}<0$ if it is relatively better quality than average or $\gamma_{j}>0$ if it is poorer quality than average. For instance, a sample with  $\rm{exp} \gamma_{\it{j}} = 4$ is four times as variable as a typical sample and will be given a quarter weight in an analysis, through the use of modified weights

(7) \begin{equation*} w_{gj}^*=w_{gj}/\exp \hat{\gamma }_j \end{equation*}

in a refit of the linear model (Equation (1)), where $w_{gj}$ are the observation-specific ‘voom’ weights. Figure 4B shows sample-specific weights ($1/\exp \hat{\gamma }_j$) estimated from the RNA-seq data shown in Figure 1B. As previously described in Ritchie *et al*. (2006) (9), either a gene-by-gene (default) or a full residual maximum likelihood (REML) scoring method can be used to fit the variance model (Equation (6)).

We generalize this approach to allow either individual sample-level variance factors (default setting; Figure 4C), or for samples to be grouped together with a different $\gamma$ for each group (referred to as a ‘block’ model; Figure 4D). For example in an experiment with six samples where the sixth sample is more variable (Figure 1A), we allow this sample to have a different variability ($\gamma_{6}$) to the remaining samples ($\gamma_{1}=\gamma_{2}=\gamma_{3}=\gamma_{4}=\gamma_{5}$). This approach can also be used when there is a logical grouping of samples based on experimental conditions, and the different conditions are expected to have different variabilities. For example in a study involving tumour and normal samples, one might observe that the tumour samples are more variable. To apply this blocked variance modelling approach, the user must define a design matrix *Z* for the variance model that reflects this grouping structure.

### Implementation

To estimate the combined weights (Equation (7)), we begin by applying the ‘voom’ procedure assuming all samples are of equivalent quality, and then use this first round of ‘voom’ weights in the variance model to obtain the $\hat{\gamma }$’s. Next, the estimated sample weights ($1/\exp \hat{\gamma }_j$) are applied in a second round of ‘voom’ to obtain observational weights that take into account variations in sample quality. The variance model (Equation (6)) is then fitted a second time and a final set of modified weights (Equation (7)) are used in the linear modelling (Equation (1)) and differential expression analysis. This approach is implemented in the voomWithQualityWeights function in the ‘limma’ package (14). This workflow has also been implemented in a Galaxy (17–19) tool available from the Galaxy Toolshed (20) to facilitate use by researchers who are unfamiliar with the R (21) programming environment.

### Methods compared

Six different analysis methods were compared on the data sets described below to determine whether particular approaches dealt better with more variable samples. These methods are:

- *No Weights:* linear modelling on the full data set where all observations are treated equally in the analysis (i.e. homoscedasticity, where no weights are specified, meaning that every expression measure is treated equally in the analysis ($w_{gj}$= 1 for all *g* and *j*). This is included to provide a measure of performance under a worst case scenario.
- *Sample Weights:* linear model analysis on the full data set with sample weights (as described in Ritchie *et al*. 2006, (9)) only (i.e. $w_{gj}$ = 1 for all *g* and *j* in Equation (7)).
- *Voom:* linear model analysis on the full data set with observational level weights (13) only (i.e. distinct $w_{gj}$ for each observation).
- *Voom + Sample Weights:* linear modelling with ‘voom’ weights combined with sample-specific weights as described in the previous section (Equation (7)).
- *Voom + Block Weights:* linear modelling with ‘voom’ weights and block weights as described in the previous section (Equation (7)). This approach estimates two or more different variabilities, one for the samples that cluster well, and additional variance factors (one or more depending on the data set) for the more variable samples. Where multiple samples with increased variation are present, each of these is assigned a distinct $\gamma_{j}$. This method assumes that the researcher can identify samples that are more variable than average via visual inspection of the MDS plots (Figure 1) or by some other method. An appropriate design matrix for the variance model must be specified.
- *Sample Removal:* discard more variable sample/(s) from the analysis (again assumes that the analyst can identify these samples somehow in advance) and apply linear models with ‘voom’ weights to the reduced data set (i.e. distinct $w_{gj}$ for each observation in the reduced data set).

In each analysis, the linear modelling (Equation (1)) was carried out on the normalized log_2_(CPM). For a given data set, the same design matrix (*X*_*g*_) was used for methods 1–5, whilst for method 6 the design matrix was reduced by the outlier sample/(s). For all methods, correction for multiple hypothesis testing was carried out using the false discovery rate (FDR) approach (22).

### Simulated RNA-seq data

Simulated data sets were generated by adapting the approach described in Law *et al*. (13) to include sample-level variability. We simulated balanced two group experimental designs where the aim is to compare gene expression between group A and group B with *n* = 3, 4 or 5 samples per group and one more variable sample in group B (sample 6). We also generated data for an unbalanced design (similar to that in Figure 1B) with *n* = 3 samples in group A and *n* = 4 samples in group B and two more variable samples, one in each group. Each simulation consisted of 10 000 genes of which 200 were DE at known FCs of 1.5, 2 or 4. The more variable samples were simulated to have increased variability of 1.2 (120%), 1.5 (150%), 2 (200%), 3 (300%), 4 (400%) or 8 (800%). A null simulation where all samples had equivalent variability (i.e. variance inflation factor = 1) was also simulated to provide an example of a well-behaved data set. The library size of the more variable sample was also varied to be 50, 60, 70, 80, 90 and 100% of the size of the other samples (2 × 10^7^ by default). Null simulations (FC = 1 for all genes i.e. no differential expression) for experiments with *n* = 3 samples per group across the range of variabilities listed above were also simulated (sample library size =100% for the more variable sample).

Baseline expression values were first generated to get a relative proportion of counts for 10 000 genes. Next the proportions were converted into expected counts by multiplying by library size and then multiplying by the desired FCs (1 (non-DE), 1.5, 2 or 4) for the first 200 genes. Negative binomial distributed counts were obtained with the specified mean and dispersion for each observation. To obtain dispersions, a trend was set to be $\psi_{gj}$ with $\psi _{gj}^{1/2}=0.12+\lambda ^{-1/2}_{gj}$ where $\lambda_{gj}$ is the expected count size. Gene-wise biological variation was generated from an inverse chi-square distribution with five degrees of freedom. The individual dispersions were set to be $\phi_{gj}=\psi_{gj}\delta_{g}$ where $5/\delta _g\sim \chi ^2_5$. For the more variable sample/s, we inflated the variability by scaling the squared gene-wise dispersions $\phi _{gj}^2$ for the particular sample/(s) *j* by a factor *k*, where *k* = 1 (equivalent variability), 1.2 (120%), 1.5 (150%), 2 (200%), 3 (300%), 4 (400%) or 8 (800%).

**Figure 2.**
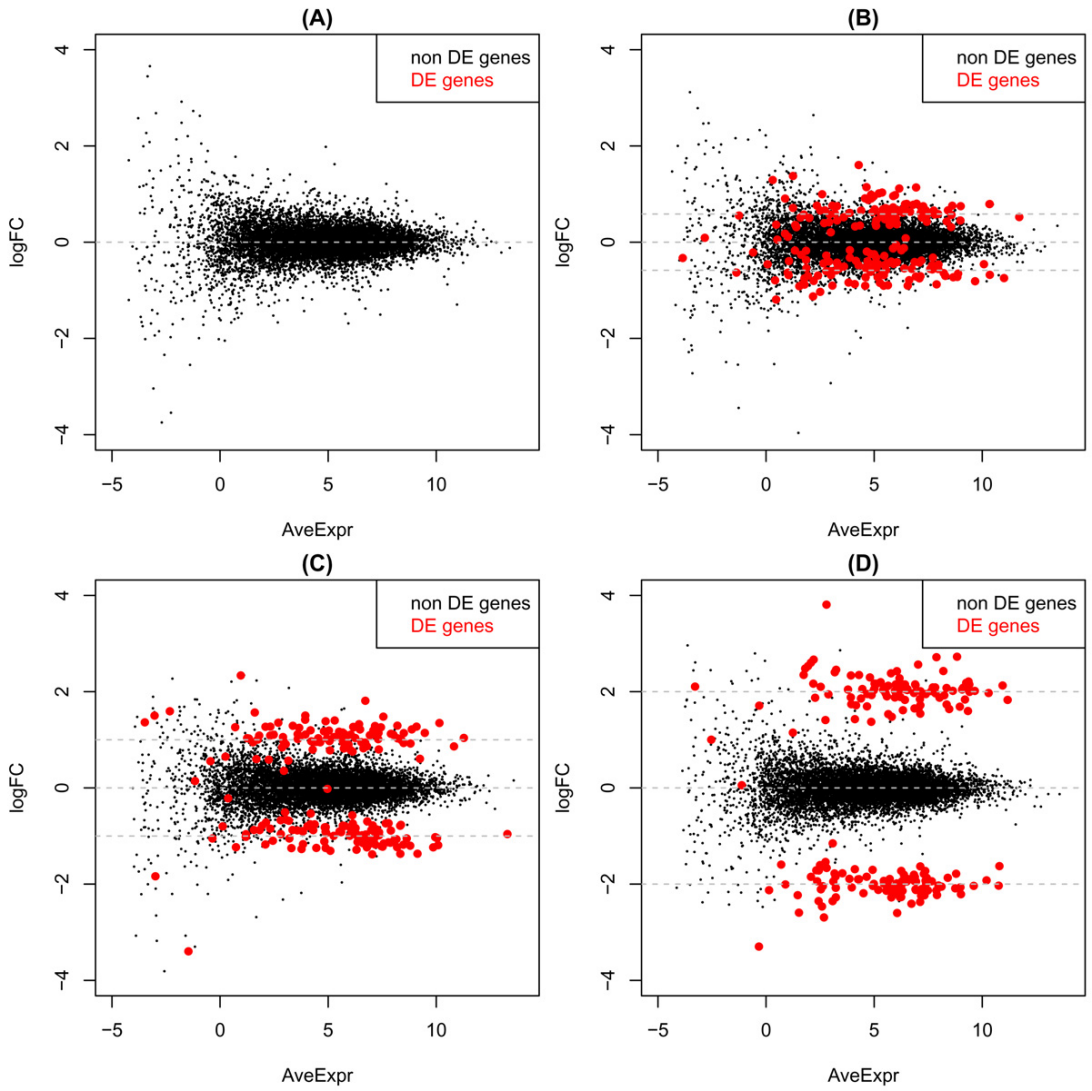
Log_2_(FC) versus average log_2_(expression level) for simulated data with 10 000 genes for samples with equivalent variability. (**A**) Null simulation with no differential expression (FC = 0 for all genes). (**B**) 200 DE genes with |FC| = 1.5. (**C**) 200 DE genes with |FC| = 2. (**D**) 200 DE genes with |FC| = 4.

**Figure 3.**
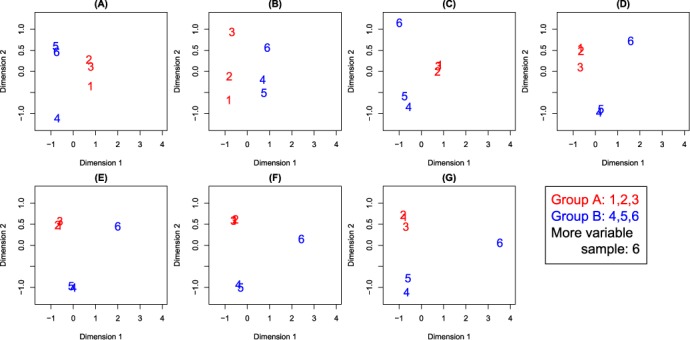
MDS plots from simulated data for different variability settings for sample 6 ranging from equivalent variability (**A**) to 120% (**B**), 150% (**C**), 200% (**D**), 300% (**E**), 400% (**F**) and 800% (**G**) more variability than the first five samples. Results shown are from a typical simulation of 10 000 genes, 200 of which have a |FC| = 4 (highlighted in red in Figure 2D).

Figure 2 shows example plots of log_2_(FC) versus average log_2_(expression level) from these simulations and Figure 3 shows representative MDS plots from the 2 group setting with *n* = 3 samples per group with equal library sizes for each sample variability setting. The position of the more variable sample (always the sixth) relative to the other samples recapitulates the separation seen in the MDS plots in Figure 1, particularly as variability increases, indicating that our simulation strategy is realistic.

In total there were 511 simulation settings (three different FCs × seven different sample variabilities × six different library sizes × four different experimental designs = 504 + seven null simulations = 511) that were each simulated 100 times. Prior to differential expression analysis, each simulated matrix of counts underwent filtering to remove genes with fewer than 10 simulated counts across all samples. Code and plots of all results are provided as ‘Supplementary Materials’.

### Control experiment

In order to provide a data set where the FCs would follow a predictable dose-response, we designed a mixture experiment (23,24) between two lung adenocarcinoma cell lines (NCI-H1975 and HCC827, both obtained from ATCC). The cell lines were chosen based on their similarity of molecular aberrations (they both bear *EGFR* mutation) and gene expression profiles compared to other lung cancer cell lines included in the Cell Line Encyclopedia (GSE3613; (25)). Cell lines from a range of passages (2–4) were grown on three separate occasions in RPMI media (Gibco) supplemented with Glutamax and 10% fetal calf serum to a 70% confluence. To replicate common experimental conditions, cell lines were treated with 0.01% Dimethyl sulfoxide (Sigma), which is commonly used as a vehicle in drug treatment experiments. After 6 h of treatment, cells were collected, snap-frozen on dry ice and stored at −80°C until required. Total RNA was extracted from between half a million and a million cells using a Total RNA Purification Kit (Norgen Biotek) with on-column DNAse treatment according to the kit instructions. RNA concentration for each pair of samples to be mixed was equalized to 100 ng/μl using Qubit RNA BR Assay Kit (Life Technologies). Replicates were pooled in known proportions to obtain mixtures ranging from pure NCI-H1975 (100:0) to pure HCC827 (0:100) and intermediate mixtures ranging from 75:25 to 50:50 to 25:75.

All mixtures corresponding to the second replicate were split into two equal aliquots. One aliquot was left intact (we refer to this as the ‘good’ replicate), whilst the second aliquot was degraded to increase variation by incubation at 37°C for 7 days in a thermal cycler with a heated lid. The RNA Integrity Number determined using TapeStation RNA ScreenTape (Agilent) was below 5 for the degraded samples and above 8 for the intact samples. This experimental design allowed us to perform an analysis on the regular samples (‘good’ analysis) and compare our results to the analysis that included the degraded samples. Ten microlitres from each replicated mixture (both good and degraded) were used for Next Generation Sequencing library preparation using Illumina’s TruSeq Total Stranded RNA with Ribozero according to the manufacturer’s instructions. Library clustering was performed on a cBot with Illumina HiSeq SR Cluster Kit v4 cBot. Libraries were sequenced as single-end 100 base pair reads at the Australian Genome Research Facility on an Illumina HiSeq 2500 with an Illumina HiSeq SBS Kit, v4. Base calling and quality scoring were performed using Real-Time Analysis (version 1.18.61) and FASTQ file generation and de-multiplexing using CASAVA (version 1.8.2). Reads from FASTQ files were aligned to the human genome (hg19) using Subread (version 1.16.1) (26) and summarized at the gene-level using the featureCounts procedure (27) and TMM normalized (3). Subsequent analysis was carried out using the ‘edgeR’ (28) and ‘limma’ (14) Bioconductor software (29). These data are available under GEO series accession number GSE64098.

### *Smchd1* experiment

RNA was extracted from 1 × 10^6^ *Smchd1+/+;EμMycTg/+* and *Smchd1MD1/MD1;EμMycTg/+* lymphoma cells using Qiagen RNeasy Minikit as per the manufacturers instructions. Libraries were prepared using Illumina’s TruSeq RNA sample preparation kit as per the manufacturers instructions and submitted to the Australian Genome Research Facility for quality control, library preparation and sequencing on the Illumina HiSeq 2000 platform using 100 base, paired end or single-end reads. Base calling and quality scoring were performed using Real-Time Analysis (version 1.17.21.3) and FASTQ file generation and de-multiplexing using CASAVA (version 1.8.2). Reads from FASTQ files were aligned to the mouse genome (mm10) using Subread (version 1.10.5) (26) and summarized at the gene-level using the featureCounts procedure (27). Subsequent analysis was carried out using the ‘edgeR’ (28) and ‘limma’ (14) Bioconductor software. The counts were transformed into CPM to standardize for differences in library-size and filtering was carried out to retain genes with a baseline expression level of at least 0.5 CPM in three or more samples. Data were TMM normalized (3) and an MDS plot was generated (Figure 1B) before linear models using various weighting strategies (described below) were fitted to summarize over replicate samples. Moderated *t*-statistics were used to assess differential expression between *Smchd1MD1/MD1* and *Smchd1+/+* (wild-type) samples, with genes ranked according to their FDR (22). These data are available under GEO series accession number GSE64099.

*Smchd1* has been shown to have a role in the regulation of clustered protocadherins and imprinted genes in diverse tissues including whole embryo, adult brain, embryonic fibroblasts, placenta and malignant and normal B cells (30–32). We obtained gene sets for these two classes of genes to use as true positives (TPs) in our analysis. To identify protocadherins, we used regular expression matching to look for this term in the gene name field of the annotation of the filtered data set, which returned eight genes (out of a total of 71 in the mouse genome). A comprehensive set of imprinted mouse genes was downloaded from http://www.mousebook.org/imprinting-gene-list and matched to the expressed genes in this data set using Gene Symbols. In total, 46 genes out of the 150 in the original list were matched.

## RESULTS

### Weighting accommodates two major sources of variability

**Figure 4.**
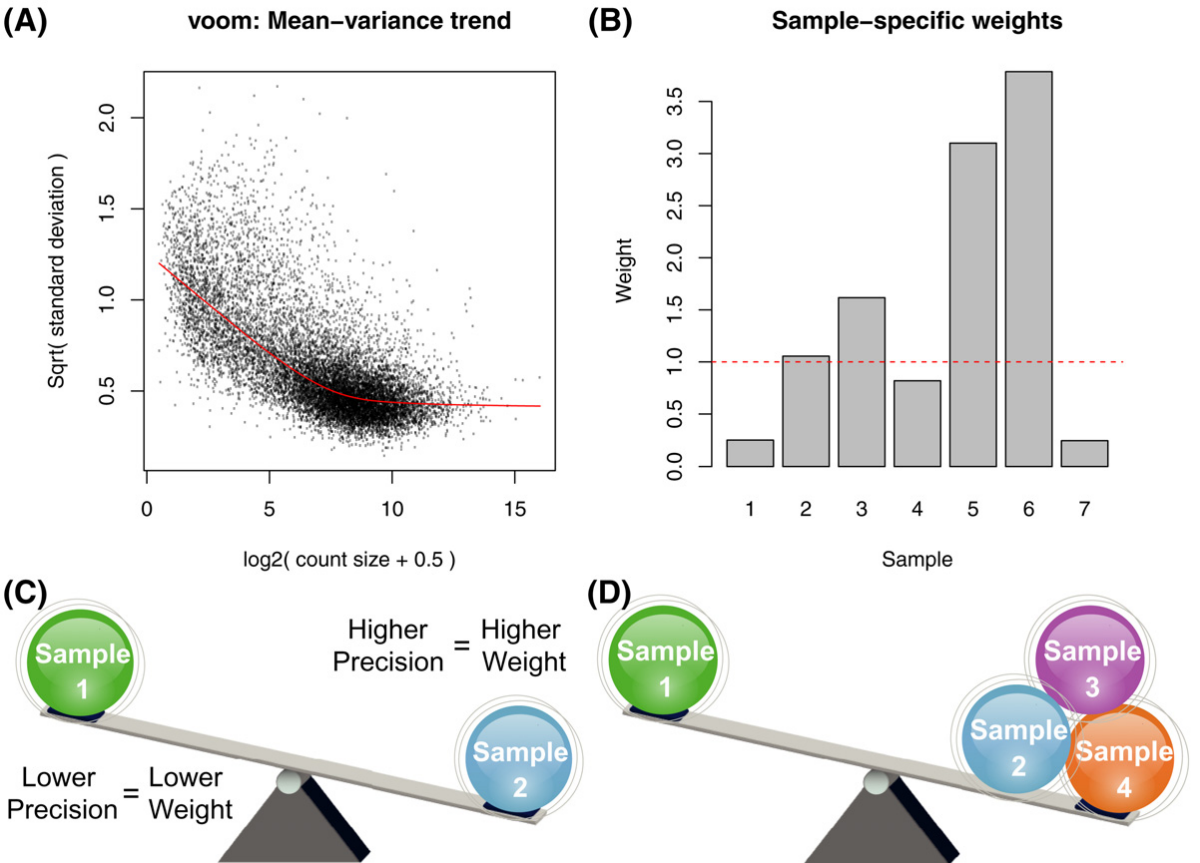
Weighting strategy where ‘voom’ weights (**A**) that model the mean-variance trend in the data and down-weight low-intensity observations are combined with sample-specific weights (**B**) or similar to model variability between different samples. Our default strategy is to model variability separately for each sample (**C**) so that each observation from a particular sample shares a common sample variance factor, which is converted into a weight (B). A second option allows samples to be grouped together (**D**) in a user-defined manner by specifying a design matrix for the variance model. We refer to this as our ‘block’ model.

The first level of variability we deal with is at the observation-level and is related to the abundance of a gene in each sample. Figure 4A shows the estimated mean–variance trend from the normalized log_2_ CPM from the *Smchd1* data set obtained using the ‘voom’ method (13) to derive observational level weights ($w$_*gj*_ i.e. a unique weight for each gene *g* in each sample *j*). Higher abundance observations tend to be more precise and are rewarded with higher relative weights whilst low abundance observations which tend to be less precise receive lower weights.

The second level of variation that we accommodate is at the sample-level as estimated using a log-linear model (the Materials and Methods section; Equation (6)). Unlike observational weights, the variance factor is shared by all genes in a given sample, meaning that a common weight particular to a given sample *j* is returned for use in the linear model analysis. We allow weights to be either distinct for each sample (default setting; Figure 4C) or to have a block structure, where certain samples share a common variance factor (Figure 4D). Figure 4B shows the relative sample weights obtained for the *Smchd1* experiment from a variance model parameterized to have sample-specific parameters. Recall from the MDS plot for this data set (Figure 1B) that samples 1 and 7 cluster less well than the other replicate samples, so down-weighting the observations from these samples in the differential expression analysis would seem sensible.

The purpose of combining sample-specific weights with ‘voom’ weights is to obtain more precise estimates of the gene expression coefficients in the linear model (Equation (1)) and improve power to detect DE genes. To assess whether this occurs, we compared the performance of this method against a number of alternatives on various simulated and experimental data sets (see the Materials and Methods section).

### Combining weights delivers the lowest FDR

**Figure 5.**
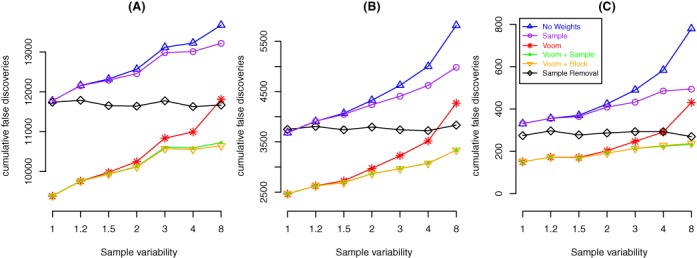
Cumulative false discoveries across 100 simulated data sets for a two-group simulation with *n* = 3 samples per group. Each panel shows results from simulations with different true positive FCs: 1.5-fold (**A**), 2-fold (**B**) and 4-fold (**C**). The y-axis shows the cumulative number of false positives amongst the top 200 genes from each analysis method across 100 independent simulations of each setting. The x-axis indicates the simulated sample variability of the sixth sample. The results from these 21 simulation settings are representative of the 511 settings explored (see ‘Supplementary Materials’ for the complete results).

Simulations allow us to increase the variation of particular samples whilst also spiking in genes at known FCs to provide us with a set of TPs. Each simulation included 200 genes out of 10 000 with particular FCs (see the Materials and Methods section and Figure 2B–D) and increased variation in particular samples (Figure 3). This configuration allowed us to assess the number of false discoveries amongst the top 200 genes identified by each method. The methods compared all used linear models to assess differential expression, with either no weights (i.e. all observations are treated equally in the analysis), sample weights, ‘voom’ weights (i.e. observational level weights only), ‘voom’ and sample weights (i.e. combined observational and sample-specific weights), ‘voom’ and block weights or an analysis with ‘voom’ weights where the more variable sample has been removed. Figure 5 shows these results for various FCs and sample variabilities cumulatively across 100 simulated data sets of each configuration for the smallest experiment (*n* = 3 samples per group, 10 000 genes). Overall the number of errors decreases for all methods as the absolute FC of the TPs increases, which is to be expected, as the problem of distinguishing DE genes from non-DE genes becomes easier for larger effect sizes (see Figure 2). Applying ‘voom’ weights in some form to the full data set (red, orange or green lines) to try and capture the simulated variability results in fewer false discoveries than either treating all observations equally in the analysis (blue lines), applying sample weights only (purple lines) or applying ‘voom’ weights to the reduced data set (black lines). The no weighting option (blue lines) and the sample weighting only method (purple lines) generally perform worst, with many more false positives relative to methods that use observational level weights. Removing more variable samples (black lines) guarantees a fairly constant number of false discoveries due to the constant noise level across the simulations (since the sixth sample is always removed), whilst for all other methods, the error rate climbs as sample variability increases. Combining ‘voom’ with sample-specific weights (green lines) or block weights (orange lines) are the best or equal best methods in all settings, producing very similar error rates to one and other. The combined ‘voom’ and sample weighting methods perform similarly to ‘voom’ alone when there is no increase in sample variability or the increase is low (120 and 150% of the regular samples) but beyond that level (200% and above) an analysis that combines ‘voom’ with sample-specific weights offers improved performance in terms of false discoveries, irrespective of the size of the simulated FCs. These results are representative of those observed for the other simulation settings examined, where library size of the more variable sample was varied or the overall size of the experiment increased (refer to ‘Supplementary Materials’).

### Combining weights increases power whilst controlling the type I error rate

**Figure 6.**
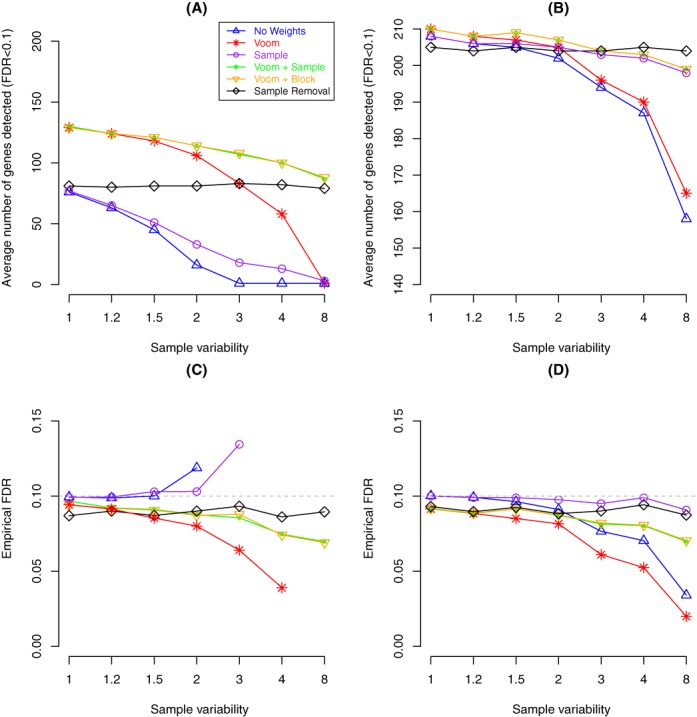
Plot assessing power (**A**,**B**) and the corresponding empirical FDR (**C, D**) at an FDR cut-off of 0.1 averaged across 100 simulated data sets. Results shown are for simulations where the TPs have |FC| = 2 (A, C) and |FC| = 4 (B, D) for various variabilities for the sixth sample (x-axis) for a two-group simulation with *n* = 3 samples per group. In panel (**C**), the empirical FDR values for sample weighting only that are off the scale are 0.42 for 400% and 0.68 for 800%. For the other methods (no weighting and ‘voom’ only), points were omitted in panel (C) when the average number of discoveries (panel (A)) was less than one gene to avoid ratios of small numbers that produce very variable FDRs. In most panels, results from combining either ‘voom’ and sample weights or ‘voom’ and block weights are over plotted as the results are the same. Boxplots of the results for each analysis method across the 100 simulated data sets generated under each sample variability setting are provided as ‘Supplementary Materials’.

We next assess the power of each method by counting the number of genes that pass an FDR cut-off of 0.1 in the different simulation settings (see the Materials and Methods section). Figure 6 shows these results averaged over 100 data sets for absolute FCs of 2 (panel (A)) and 4 (panel (B)), respectively (results for an FC of 1.5 were omitted as all methods lacked power, making no or very few discoveries irrespective of the variability level of the sixth sample). In almost all situations, the methods with the greatest power are ‘voom’ combined with either sample (green lines) or block weights (orange lines). Removing the more variable sample (black line) is marginally more powerful for a simulated FC of 4 when the sixth sample is ≥400% more variable; however, in general we see that there is a penalty to be paid for leaving out data when the sample size is small. In this simulation, sample removal means the data set is 1/6 (16.7%) smaller, systematically reducing power relative to the weighted methods that make use of the full data set. Methods that use all samples lose power as the variability of the sixth sample increases, with no weighting (blue lines) losing out most rapidly followed by either sample weighting only (purple lines, panel (A)) or ‘voom’ only (red lines, panel (B)).

The empirical FDRs for each method on the same data using the same cut-off (FDR < 0.1) is also shown in Figure 6 (simulations with FCs of 2 in panel (C) and FCs of 4 in panel (D)). In most situations, fewer than 10% of the discoveries made are errors. When the average number of discoveries made falls below 50 in simulations where the noise level increases as seen for the no weighting or sample weighting only options when sample variability reaches 200% (blue or purple line, panel (A)) the empirical FDR starts to climb (panel (C)). In the case of the sample weighting alternative, it rises above the level of guessing (empirical FDR = 0.68 at 800%). In the scenario where removing the more variable sample offered marginal improvement in power relative to the sample weighting methods (panel (B), sample variability ≥400%), we see that the FDR (panel (D)) is marginally lower for the combined ‘voom’ and sample weighting alternatives, meaning that the results obtained from the combined weighting approach will contain fewer errors than the results obtained from either removing the more variable sample or applying sample weights only. Similar results were obtained using an FDR cut-off of 0.05 (see ‘Supplementary Materials’).

**Figure 7.**
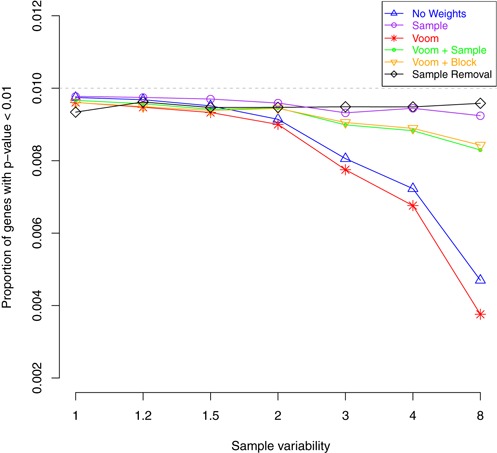
Average type I error rates from null simulations (FC = 1 for all genes) using a *P*-value cut-off of 0.01 from 100 data sets with *n* = 3 samples per group. All methods control the false discovery rate at this level, irrespective of the simulated variability of the sixth sample (x-axis). ‘Voom’ on the full data set (red line) and not using weights (blue line) becomes increasingly conservative as sample variability increases.

We also examined how the different alternatives control the ‘Type I’ error rate in a simulation where there is no differential expression (i.e. FC = 1 for all genes; Figure 2A) between the two groups (Figure 7). In this situation, the *P*-values should have an approximately uniform distribution, meaning that the expected proportion of *P*-values below a certain cut-off should be less than or equal to this value. For this analysis we use the raw *P*-value cut-off of 0.01 (grey dashed line) and plot the proportion of genes that have a *P*-value less than this threshold. The sample removal method (black line) depicts results from applying ‘voom’ alone when the noise level is constant and, as we would expect, has a fairly constant type I error rate. Across a range of variability inflation factors for the sixth sample, the new weighting methods approximately hold their size, even when this sample is eight times more variable than the others, being only slightly more conservative than removing the sample altogether. Treating all observations as equal (no weights) or using ‘voom’ on the complete data set becomes gradually more conservative as sample variability increases, reporting around half as many genes than would be expected by chance when the more variable sample is eight times as variable as the other samples.

The results from our simulations are compelling. By combining observational and sample-specific weights we make fewer false discoveries and have greater power than applying either sample weights alone or ‘voom’ alone (on either the full data set or on a subset of the data set after the more variable sample has been removed). Ignoring the simulated variability and treating all observations equally or applying sample weights only delivers the poorest results. We therefore exclude these two alternatives from subsequent analyses. Whilst these simulations were intended to approximate RNA-seq data and reflect the variations observed in practice, we now shift our focus to the analysis of the ‘Control’ and the *Smchd1* experiments to assess whether our modelling approach is useful in practice.

### Degraded samples are down-weighted by our method

**Figure 8.**
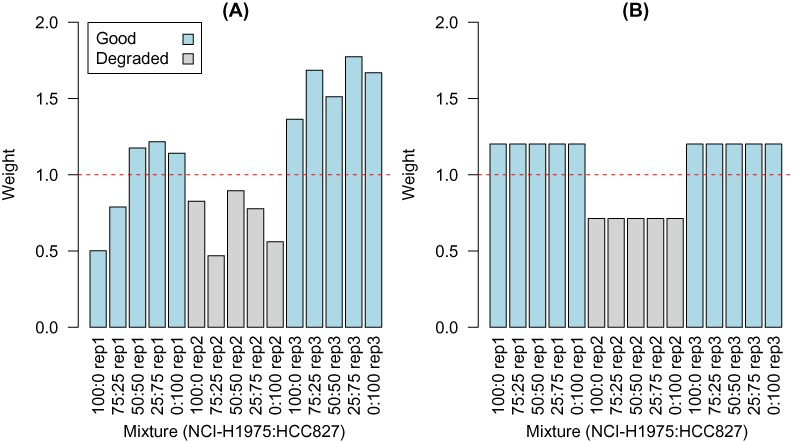
Degraded RNA samples (replicate 2, shaded in grey) from the control experiment are correctly assigned lower weights by the combined ‘voom’ and sample weighting procedure (**A**), with an average weight of 0.70 across these five samples, compared to an average of 1.28 for the non-degraded samples (replicates 1 and 3, shaded in blue). A similar result is obtained for block weighting (**B**), with a weight of 0.71 assigned to the five degraded samples versus 1.20 for the remaining samples. When ‘voom’ was combined with sample weighting on the good samples, the weights were equivalent for the replicate 2 samples (1.06) and the remaining samples (1.07, data not shown).

To simulate variations in sample quality experimentally, we performed a control experiment where particular samples were mistreated by applying temperature (37°C for 7 days; see the Materials and Methods section). The degraded samples were successfully detected by our combined ‘voom’ and sample-specific weighting method and down-weighted in the analysis (Figure 8A). Applying ‘voom’ with block weights, which allowed these samples to have a distinct weight relative to the remaining samples, gave a similar result, with systematically lower weights assigned to these five samples (Figure 8B).

Table 1 summarizes the effect of applying these weights in a differential expression analysis. The number of DE genes and the recall of genes identified as DE in the ‘voom’ analysis of the good data set that were also identified in the analysis based on the degraded replicate 2 samples are presented. As the mixtures compared get more similar, the overall number of genes detected by each method decreases, as we would expect. The use of ‘voom’ with sample weights (column 3) or ‘voom’ with block weights (column 4) recovers more genes than competing methods, such as ‘voom’ only (column 2). The genes identified were also more consistent with those recovered from the analysis based on the good quality samples. Removing the more variable samples (column 5) recovers fewer genes that are less concordant with the results from analysing the good samples. This again highlights the serious loss of power that results when we remove samples from a small experiment, which in this case leaves us with only two replicates out of three (i.e. we lose 1/3 of the data) to perform inference on.

**Table 1.** The number of DE genes (FDR < 0.05) for various comparisons from the control data set

Mixture Comparison	Voom	Voom + Sample	Voom + Block	Sample Removal	‘Good’ Analysis
100:0 versus 0:100	11 403 88.7%	11 872 **90.4%**	11 662 **90.4%**	10 534 82.7%	12 262
75:25 versus 0:100	9692 84.8%	10250 **87.2%**	9936 86.8%	8592 76.6%	10 773
50:50 versus 0:100	6924 78.1%	7441 **81.5%**	7152 80.8%	5650 65.6%	8212
25:75 versus 0:100	2479 64.0%	2524 64.6%	2604 **67.3%**	1664 44.8%	3430

Columns 2–4 show results obtained using the degraded replicate 2 samples, column 5 shows results after removing these degraded samples, and the final column shows results from a ‘voom’ analysis using the ‘good’ samples only (i.e. non-degraded replicate 2). The percentages shown are the number of genes that overlap with the genes identified using the good data. The highest recall % is highlighted in bold.

### Combining weights delivers biologically meaningful results

In this second example, we analyse data from an RNA-seq experiment that aimed to identify genes that are transcriptionally regulated by *Smchd1* in lymphoma cell lines. This experiment consisted of three wild-type and four mutant samples (see the Materials and Methods section). Table 2 summarizes the results from this analysis. Applying observational level weights (‘voom’ alone) on the full data set gives very few DE genes (12) at an FDR cut-off of 0.05. The removal of the two more variable samples identified by visual inspection (samples 1 and 7; Figure 1B) followed by ‘voom’ or applying ‘voom’ with block weights recovers ∼500 genes, whilst combining ‘voom’ with sample-specific weights (Figure 4B) discovers the most genes with ∼1500 (i.e. three times as many as the next best alternative). It also assigned the highest significance to *Smchd1*, the gene that was mutated in this study, with an FDR of 1.98 × 10^−5^, closely followed by ‘voom’ with block weights (5.14 × 10^−5^), then ‘voom’ on the full data set (2.66 × 10^−4^) and lastly ‘voom’ after sample removal (4.28 × 10^−3^).

**Table 2.** Total number of DE genes (FDR < 0.05) from different analyses of the *Smchd1* data set and *P*-values from ROAST gene set testing for gene sets that are known to be regulated by *Smchd1*

Gene set	Voom	Voom + Sample	Voom + Block	Sample Removal
				
Protocadherins	0.0581	**0.00614**	0.0235	0.0707
Imprinted genes	0.0594	**0.0198**	0.0607	0.337
Total DE genes	12	1478	488	492

The lowest *P*-value is highlighted in bold.

The two gene signatures that have been reported to be regulated by *Smchd1* in previous studies (the Protocadherins and a subset of Imprinted genes) (30–32) were tested for up-regulation using the ROAST (33) gene set test, which incorporates weights as part of its testing procedure. We test for up-regulation of all expressed Protocadherins and imprinted genes since we are comparing samples where *Smchd1* has been lost (*Smchd1MD1/MD1*) against wild-type (*Smchd1+/+*) samples and expect genes directly regulated by *Smchd1* to increase in expression under this comparison. The *P*-values in Table 2 are from a directional ROAST analysis for each method. The smallest *P*-values for up-regulation (Table 2) are obtained through the combined application of ‘voom’ and sample weights. Thus not only does the joint modelling of observational and sample-specific variation discover more genes, it improves the recovery of known gene sets regulated by *Smchd1*, indicating that these extra discoveries are likely to be biologically meaningful rather than noise. We have successfully applied this approach on a number of other RNA-seq data sets and achieved similar results (data not shown).

## DISCUSSION

We have shown that modelling heteroscedasticity at both the observational and sample-level can enhance the results of an RNA-seq differential expression analysis. Simulations demonstrated that combining ‘voom’ with sample-specific weights can lead to a more powerful analysis with a low FDR relative to other alternatives, such as either ‘voom’ or sample-specific weighting on their own. The analysis of data from a specially designed control experiment and a gene mutation study that each contained more variable samples showed us that the extra discoveries made by our weighting strategy were likely to be biologically meaningful, as they were either in better agreement with the results obtained from analysing a clean version of the same data set or better able to recover known gene signatures. The weights derived by this approach are propagated through each step of a ‘limma’ analysis, including gene set testing.

Although we have demonstrated this approach using simple two group experimental designs with varying sample sizes, in practice more complicated experimental designs can be accommodated just as easily. Our method also allows flexibility in the structure of the sample-level variance model. The default sample-specific mode is recommended in most situations as it offers excellent performance and does not require any special input from the user. Where more information is available, the user is free to specify other configurations, such as a block structure where the more variable samples are given distinct weights relative to the remaining samples. This approach relies on the data analyst being able to identify suspect samples in advance of fitting the model, which may not always be possible. A further possibility that this approach allows that we have yet to explore is the modelling of group-specific differences in variability (e.g. tumour versus normal).

We have intentionally focused on the performance of these methods on small data sets, as these are not only the most common in our experience, but also where the most gains can be made by modelling the residuals ‘between’ genes to get a handle on the sample-level variation that is present. For larger experiments, the usefulness of other approaches such as robust methods (34) that can determine unusual observations by looking at the residuals ‘within’ each gene would be expected to come to the fore.

It is important to note that the minimum sample size where this approach would be recommended is an experiment with three or more samples per group. Theoretically, the variance model can be fitted in smaller studies with a minimum of two samples per group. When a block model is fitted, the software will return block-wise weights even when some or all of the groups have fewer than three samples. When a sample-specific model is fitted, both samples in a group of size 2 will be assigned equal weight (i.e. equivalent to block weights for that group), which may or may not be desirable. In an experiment with three samples in one group and two in a second, combined ‘voom’ and sample-specific weights could be beneficial, so long as the more variable sample belongs to the larger group.

One obvious consequence of a more powerful analysis through combining ‘voom’ and sample-specific weights is that it may result in a large number of genes passing a given FDR cut-off. In situations where there is a need to further refine the list of genes, we recommend the user apply a test for a given FC cut-off using the TREAT method (16).

We anticipate that this approach may also be useful for dealing with more variable samples in differential binding analyses for chromatin immunoprecipitation sequencing studies or single cell transcriptomic profiling experiments, when the goal is to summarize over replicate samples and look for differences between experimental groups. Further work will be to adapt this approach for incorporation in edgeR (28), which can also accommodate weights (34) in its generalized linear modelling framework (7).

## AVAILABILITY

The weighting methods described in this paper are implemented in the voomWithQualityWeights function in the open-source ‘limma’ package distributed as part of the Bioconductor project (http://www.bioconductor.org/). A Galaxy tool that includes the option to apply ‘voom’ with sample-specific weights in an RNA-seq differential expression analysis is available from the Galaxy Toolshed at https://toolshed.g2.bx.psu.edu/view/shians/voom_rnaseq. The R code and plots of results for all simulation settings along with the R code to carry out the analyses of the ‘Control’ and ‘Smchd1’ RNA-seq experiments are provided as ‘Supplementary Materials’ at http://bioinf.wehi.edu.au/voomWithQualityWeights/. Experimental data are accessible through GEO series accession numbers GSE64098 and GSE64099.
